# Clinical Screening for Lower Limb Muscle Weakness in Community-Dwelling Older Women

**DOI:** 10.1093/geroni/igaa057.742

**Published:** 2020-12-16

**Authors:** Daniela Abreu, Jaqueline Porto

**Affiliations:** 1 University of Sao Paulo, Ribeirao Preto, Sao Paulo, Brazil; 2 Ribeirão Preto School of Medicine of the University of São Paulo, Ribeirão Preto, Sao Paulo, Brazil

## Abstract

The objective of the present study was to evaluate the ability of the five times sit to stand test (5TSST), grip strength (GS) and step test (ST) to detect older women with reduced lower-limb muscle strength (LLMS), and to investigate the clinical usefulness of the combination of such tests. One hundred and nineteen older women were submitted to the 5TSST, GS, ST and lower limb peak torque by an isokinetic dynamometer. The capacity of the clinical tests to detect older women with reduced LLMS was measured using the ROC curve, followed by calculation of posttest probability (PoTP). The results show that a ST score of 0.24 cm per cm of participant’s height shows the best PoTP for a positive test (72%). However, the combination of the ST and 5TSST enhances the accuracy from 48% (prevalence of weakness in the population) to 82.6% if both tests are positive, and decreases the PoTP from 48% to 11.4% if both tests are negative. The inclusion of GS provided additional benefits of small magnitude. In conclusion, the ST performed alone or in combination with 5TSST could be an alternative for clinical screening of LLMS reduction in older women. The early identification of impairment of lower-limb muscle strength in independent older adults may favor early intervention and prevention of negative outcomes such as falls and functional limitations.

